# The correlation between auditory event-related potentials characteristics and cognitive function in insular glioma patients

**DOI:** 10.3389/fneur.2025.1692484

**Published:** 2026-01-12

**Authors:** Chuanhao Zhang, Xinxin Wang, Bowen Xue, Xinyu Song, Qifeng He, Xinlong Liu, Zhenghai Deng, Ruquan Han, Zonggang Hou, Jian Xie

**Affiliations:** 1 Department of Neurosurgery, Beijing Tiantan Hospital, Capital Medical University, Beijing, China; 2 Department of Anesthesiology, Beijing Tiantan Hospital, Capital Medical University, Beijing, China; 3 China National Clinical Research Center for Neurological Diseases, Beijing, China

**Keywords:** auditory event-related potentials, event-related spectral perturbation, glioma, insular, mismatch negativity, neuropsychological, P300, theta oscillations

## Abstract

**Background:**

Auditory Event-Related Potentials (AERPs) characteristics, including mismatch negativity (MMN), P300, and event-related spectral perturbation (ERSP) are useful psychophysiological index that reflects cognitive functions, however, the relationship between AERPs characteristics and neuropsychological tests scores in insular glioma patients is unclear.

**Methods:**

Twenty-one insular glioma patients and 17 healthy control (HC) subjects were recruited. Age and education level were matched between the two groups. A correlation analysis was conducted between AERPs parametersr and neuropsychological test scores in patients with insular glioma.

**Results:**

The P300 peak latency at Fz electrodes under deviant stimuli showed significant negative correlations with execution function, fluency, calculation, and visual perception; and it showed significant positive correlations with Hamilton Depression Rating Scale (HDRS) scores, Hamilton Anxiety Rating Scale (HAMA) scores and Beck Depression Inventory (BDI) scores. The P300 peak latency at Cz electrodes under deviant stimuli showed significant negative correlations with fluency; and it showed significant positive correlations with HAMA scores and BDI scores. The MMN mean amplitude at Fz electrodes under novel stimuli showed significant positive correlations with fluency. Theta power at F4, F1, C1, AF3, and AF4 electrodes is positively correlated with naming function, while theta power at Fz electrode is negatively correlated with anxiety and action.

**Conclusion:**

The P300 peak latency and MMN mean amplitude are associated with multiple cognitive functions insular glioma patients, and event-related theta power mainly associated with naming function of insular glioma patients. Our results suggest that P300 latency, MMN amplitude and event-related theta power could be used as biological markers that indicate impaired neuropsychological functions in insular glioma patients.

## 1 Introduction

Glioma, the most common form of tumor in the central nervous system, has highly infiltrative feature, and surgical resection holds a pivotal role (1). The insula is recognized as a prime site for glioma formation, comprising 25% of low-grade gliomas and 10.8% of glioblastomas multiforme (GBMs) in the supratentorial region (2).

Insula plays an important role in cognitive function. It participates in processes such as memory, decision-making, and time perception (3). The front-end of insula compares the integrated information with personal past experiences and memories to support advanced cognitive functions such as consciousness, decision-making, and cognitive control. In addition, insula also participates in the handling of positive and negative emotions, including happiness, sadness, fear, disgust, anxiety, etc. (4). Preoperative cognitive function testing can inform prognosis and post-operative planning for insular glioma patients, and impact intraoperative strategy to some extent. At present, the detection of cognitive level in patients with insula glioma relies on neuropsychological test scores, however, due to the limited testing time of most scales, subjective factors of the rater, and factors such as the patient’s emotional state, education level, and cultural background may affect the test results (5).

Event-related potentials (ERPs) constitute bioelectrical responses detected subsequent to the administration of a specific stimulus to the nervous system, reflecting the higher cortical brain’s response to a particular stimulus (6). Among the ERPs elicited by various sensory stimuli, auditory ERPs (AERPs) have been extensively utilized in studies assessing brain information processing capabilities (7). P300 and Mismatch negativity (MMN) are the most prevalent components of AERPs. P300 is a positive potential that peaks approximately 300 milliseconds after stimulus onset and is associated with various conscious activities, including attention allocation, sustained attention, working memory updating, and information classification (8–11). MMN objectively reflects the automatic processing of auditory information in the brain and serves as a proxy for auditory memory integrity (12).

Event-related spectral perturbations (ERSPs) serve as a approach for detecting neural oscillations. They are capable of revealing task-associated neuromodulation across various frequency bands. Neural oscillations occurring at different frequencies are linked to a wide range of cognitive functions (13). Prior research has indicated a notable connection between the theta band oscillations, especially occurring in the frontal cortex, and cognitive workload (14). In particular, neural oscillations within the theta and alpha frequency bands are correlated with cognitive control during the Go/Nogo task (15–17). Fluctuations in theta activity play a crucial role in engaging cognitive control mechanisms associated with both response execution and inhibition (18).

There is no study that have systematically investigated the relationship between neuropsychological tests and AERPs characteristics of insular glioma patients. Previous studies have shown that neuropsychological tests such as overall cognitive and executive function in patients with Parkinson’s disease, Alzheimer’s disease and aphasia are well correlated with P300 latency and amplitude, patients with poor cognitive and executive functions have longer P300 latency and higher P300 amplitude (19, 20). Moreover, in patients with insomnia, the MMN amplitude is associated with cognitive dysfunction and the MMN latency is significantly correlated with depressive tendency, patients with a tendency toward depression have a longer MMN latency period (21). In the study of neural oscillation in patients with prolactinomas, in Go trials, a negative correlation was identified between theta power and participants’ reaction times while a positive correlation was observed between theta power and the hit rate. This indicates that heightened theta activity in the Go condition is associated with improved task performance. In the Nogo condition, individuals exhibiting stronger theta activity demonstrated a reduced tendency to make false alarms (22).

This study aimed to compare AERPs characteristics between insular glioma patients and normal matched controls and conduct a correlation analysis between AERPs characteristics and neuropsychological test scores in patients with insular glioma to explore the possibility of combining AERPs characteristics with neuropsychological test results to more accurately assess insular glioma patients’ cognitive status.

## 2 Methods

### 2.1 Participants

In this study, a total of 21 insular glioma patients (PT) were recruited from Beijing Tiantan Hospital. All the patients were at their first diagnosis of unilateral insular gliomas without adjuvant oncological therapies and did not have additional neurological or psychiatric disorders. The clinical data and patient demographic were retrieved from the medical records of our hospital. In addition, this study recruited 17 healthy controls (HC) and required them to complete questionnaires on basic information and health status. Based on the answers, individuals with chronic disease risk, drug dependence, history of mental illness, cardiovascular disease, hypertension, stroke and hearing impairment were excluded. These patients and HC were confirmed to be right-handed users through the Edinburgh Chiral Questionnaire. This study was approved by the Institutional Review Board of Beijing Tiantan Hospital, all patients and HC signed informed consent forms.

The patient inclusion criteria are: aged 18–60 years; the first diagnosis of insular gliomas (WHO grade 2–4), and the main body of the lesion is located in the entire insula; the gliomas are unilateral; Mother tongue of patients is Chinese; participate voluntarily and be able to follow the research schedule and testing procedures; participate had no seizures in the month prior to ERP measurement.

The patient exclusion criteria are: patients with additional neurological or psychiatric disorders, infectious diseases, hypertension, diabetes and other serious systemic diseases; patients received adjuvant oncological therapies; Hearing impairment; Long term smoking, drinking, or taking psychotropic drugs; Leftist hand.

### 2.2 Neurocognitive function test

This study evaluates the cognitive status of patients through the Montreal Cognitive Assessment (MoCA) score. The MoCA scale serves as a validated instrument tailored for the swift screening of mild cognitive impairment and early-stage Alzheimer’s disease, encompassing diverse cognitive domains such as attention, memory, language, executive function, visual–spatial skills, abstract thinking, computational abilities, and orientation. Prior to undergoing MoCA testing, all participants were thoroughly instructed regarding the purpose and methodology of the assessment. The evaluation was conducted by a solitary researcher in a tranquil and undisturbed setting to guarantee the accuracy and uniformity of the results. The duration of the evaluation for each participant ranged approximately from 10 to 15 min, with slight variations contingent upon the subject’s reaction speed and processing capabilities. The MoCA scale carries a total score of 30 points, and scores beneath 26 points (with adjustments made for educational attainment) are commonly interpreted as indicative of potential cognitive decline. It is imperative to mention that for participants with fewer than 12 years of education, an extra point is appended to their raw scores for adjustment purposes, accounting for the influence of educational level on cognitive evaluation.

We utilized the Hamilton Anxiety Rating Scale (HAMA) and Hamilton Depression Rating Scale (HDRS) as assessment instruments to gage patients’ levels of depression and anxiety. These two scales are internationally acknowledged and extensively employed as standardized tools for assessing the intensity of anxiety and depression symptoms in both clinical practice and research endeavors. The Hamilton Anxiety Scale (HAMA) comprises 14 items intended to evaluate an individual’s anxiety symptoms experienced over the past week, encompassing both psychological and physical manifestations. Each item is rated according to the severity of symptoms, spanning from 0 (indicating no symptoms) to 4 (indicating severe symptoms). The cumulative score provides a comprehensive reflection of the overall severity of anxiety, with higher scores signifying more pronounced levels of anxiety.

The Hamilton Depression Rating Scale (HDRS) typically encompasses versions with 17 or 21 items, designed to evaluate the intensity of depressive symptoms experienced over the past week. This scale encompasses numerous facets of depression, such as low mood, insomnia, diminished interest in work and activities, and more. Analogous to the HAMA, each item is rated according to the severity of symptoms, with a higher aggregate score signifying a more pronounced level of depression.

The Beck Depression Inventory (BDI) serves to assess and quantify the presence and severity of depressive symptoms in patients with brain tumors. Developed by Aaron T. Beck et al., this self-assessment scale is rooted in the clinical manifestations of depression and is widely employed in both clinical practice and research contexts for the evaluation and monitoring of depressive symptoms. The BDI comprises 21 items, each of which describes specific depressive symptoms or attitudes, such as sadness, pessimism, insomnia, fatigue, and self-evaluation. Each item is scored on a scale ranging from 0 (indicating no symptom presence) to 3 (indicating high severity), yielding a total score that can range from 0 to 63 points. A higher total score signifies more severe depressive symptoms. Prior to administering the BDI assessment, all participants were provided with comprehensive assessment instructions to guarantee that they fully comprehended each question and responded based on their genuine feelings experienced over the past week.

### 2.3 EEG recording

EEG signals were recorded using 64 channel scalp electrodes (Model: BrainAmp^MR^, Brain Products GmbH, Germany), with an Ag–AgCl cap. The online sampling rate was 1,000 Hz, and the resistance was controlled below 20 k*Ω* for each electrode (most were below 10 K Ω). CPz was set as a reference electrode during recording.

AERP measurement is performed using auditory stimulus events based on the Oddball paradigm, with the following three types of sound stimuli: Standard stimuli (Std), 500 Hz pure tone, lasting for 100 ms, with a probability of occurrence of 70%; Deviant stimuli (Dev), 1,000 Hz pure tone, lasting for 100 ms, with a probability of occurrence of 15%; Novel stimuli (Nov), a computer-generated pronunciation of a patient’s name, with a probability of occurrence of 15%. The above three stimuli are used a total of 400 times, with an interval of 800 to 1,200 ms and a duration of about 8 min. The above stimulus information was presented in a pseudo-random manner using E-prime3.0 software (Psychology Software Tools, Pittsburgh, PA). The deviant and novel stimuli were preceded by a minimum of two consecutive standard tones. The auditory stimulus was presented via a pair of Sennheiser CX 80S earphones, with the volume adjusted to a comfortable level for the subjects, ranging between 60 and 80 dB.

### 2.4 EEG preprocessing

Preprocess raw EEG data using the EEGLAB toolkit based on MATLAB environment. Continuous EEG data is band-pass filtered at 1–30 Hz. Segment each trial using a time window of 1,500 ms (−500–1,000 ms) and perform baseline correction using the pre stimulus interval. Afterwards, visual inspection was conducted on the segmented data, and independent component analysis (ICA) was used to correct the samples contaminated by eye blinking and body movement, with the average value of bilateral mastoid as a re-reference.

### 2.5 Statistical analysis

Each trial of each subject was overlayed and averaged according to different stimulus types to obtain the average AERP waveform between trials. Subsequently, the average AERP waveforms of all subjects were averaged again to obtain group level AERP waveforms, and the scalp topography distribution was calculated. MMN is defined as the negative component generated in the differential wave of Dev minus Std or Nov minus Std within 150–250 ms after stimulation. P300 is the positive component generated in the differential wave within 300–500 ms after stimulation. MMN and P300 were both measured on the Fz and Cz electrode. Calculate the average wave amplitude and peak latencies of MMN and P300 components separately within the above time window and electrode.

To detect neural oscillations linked to the processing of distinct auditory stimuli, the time-domain data from the AERP was converted into the time-frequency domain using a short-time Fourier transform. The spectrograms underwent correction for each frequency by employing subtraction, utilizing a reference interval spanning from −400 to −100 ms relative to the onset of the auditory stimulus. Subsequently, after identifying the frequency band of interest through a comprehensive whole-brain time-frequency analysis, we proceeded to examine the differences between the two groups specifically within that frequency band.

Statistical analyses were conducted using SPSS version 23.0 (IBM Corp., Armonk, NY, United States). For continuous variables, independent samples t-tests were employed for group comparisons. When data distributions deviated significantly from normality (as assessed by Shapiro–Wilk tests or graphical evaluation), non-parametric Mann–Whitney U tests were utilized instead. The Spearman correlation was used to analyze the relationships between the neurophysiology indicators and the neuropsychological test scores. All statistical tests were two-tailed, and *p*-value < 0.05 was considered statistically significant.

## 3 Results

### 3.1 Clinical and demographic characteristics

21 insular glioma patients and 17 HC were recruited in the study. Among them, 9 patients had insular gliomas located on the left side, and 12 patients had insular gliomas located on the right side. And all patients had their entire insular lobe invaded on one side. There is no difference in age, gender, BMI and education level between the patients and healthy control (all *p* > 0.05; Table 1).

**Table 1:** Clinical characteristics and demographic features of participants.

Groups	Sex (male)	Age	BMI	Education level (year)
Patients	14	41	24.86	13.1
Healthy Controls	11	37.76	23.85	12.76
P	0.903	0.25	0.401	0.772

### 3.2 Amplitude and latency of P300 and MMN

**Figure 1:**
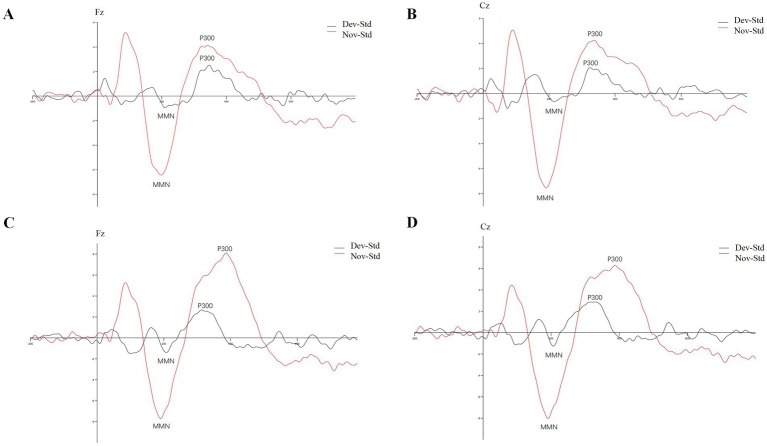
Average MMN and P300 waveforms at the Fz and Cz electrodes from insular glioma patients and healthy controls after different type of stimuli: **(A)** insular glioma patients at Fz electrode; **(B)** insular glioma patients at Cz electrode; **(C)** healthy controls at Fz electrode; **(D)** healthy controls at Cz electrode.

The obtained average MMN and P300 waveforms at the Fz and Cz electrodes from insular glioma patients and HC after different type of stimuli were presented in Figure 1. The P300 mean amplitude of insular glioma patients induced by novel stimuli at the Fz electrode was significantly lower than that of the HC (*p* = 0.017), the P300 peak latency of insular glioma patients at the Fz electrode and at the deviant stimuli Cz electrode were longer than those of the HC. The MMN peak latency of insular glioma patients at the deviant stimuli Fz electrode were longer than that of the HC (*p* = 0.02; Table 2).

**Table 2:** Amplitude and latency of P300 and MMN of participants.

Indicator	PT	HC	*p*
P300Fz L-DEV	378.76 ± 61.58	349.41 ± 47.41	0.125
P300Fz L-NOV	384.05 ± 65.19	376.18 ± 44.65	0.671
P300Cz L-DEV	381.10 ± 56.21	348.12 ± 43.52	0.061
P300Cz L-NOV	372.24 ± 46.65	381.35 ± 48.03	0.569
MMNFz L-DEV	191.81 ± 29.26	170.82 ± 20.38	0.02*
MMNFz L-NOV	194.57 ± 33.47	211.94 ± 34.37	0.135
MMNCz L-DEV	191.90 ± 30.63	190.82 ± 35.58	0.923
MMNCz L-NOV	198.90 ± 38.29	215.88 ± 30.20	0.155
P300Fz A-DEV	1.09 ± 1.84	0.35 ± 1.33	0.183
P300Fz A-NOV	2.81 ± 2.46	5.17 ± 3.18	0.017*
P300Cz A-DEV	0.84 ± 2.05	0.57 ± 1.19	0.649
P300Cz A-NOV	2.87 ± 3.09	4.45 ± 3.29	0.148
MMNFz A-DEV	−0.22 ± 1.57	−0.17 ± 2.41	0.937
MMNFz A-NOV	−4.83 ± 2.89	−5.37 ± 3.65	0.622
MMNCz A-DEV	0.19 ± 1.46	0.16 ± 2.28	0.972
MMNCz A-NOV	−5.43 ± 2.76	−5.85 ± 3.28	0.679

P300Fz L-DEV, P300 latency induced by deviat stimuli at the Fz point; P300Fz L-NOV, P300 latency induced by novel stimulil at the Fz point; P300Cz L-DEV, P300 latency induced by deviat stimuli at the Cz point; P300Cz L-NOV, P300 latency induced by novel stimulil at the Cz point; MMNFz L-DEV, MMN latency induced by deviat stimuli at the Fz point; MMNFz L-NOV, MMN latency induced by novel stimuli at the Fz point; MMNCz L-DEV, MMN latency induced by deviat stimuli at the Cz point; MMNCz L-NOV, MMN latency induced by novel stimuli at the Cz point; P300Fz A-DEV, P300 amplitude induced by deviat stimuli at the Fz poin; P300Fz A-NOV, P300 amplitude induced by novel stimuli at the Fz point; P300Cz A-DEV, P300 amplitude induced by deviat stimuli at Cz point; P300Cz A-NOV, P300 amplitude induced by novel stimuli at the Cz point; MMNFz A-DEV, MMN amplitude induced by deviat stimuli at the Fz point; MMNFz A-NOV, MMN amplitude induced by novel stimuli at the Fz point; MMNCz A-DEV, MMN amplitude induced by deviat stimuli at the Cz point; MMNCz A-NOV, MMN amplitude induced by novel stimuli at the Cz point. *p < 0.05.

### 3.3 Whole-brain time–frequency analysis of all patients

**Figure 2:**
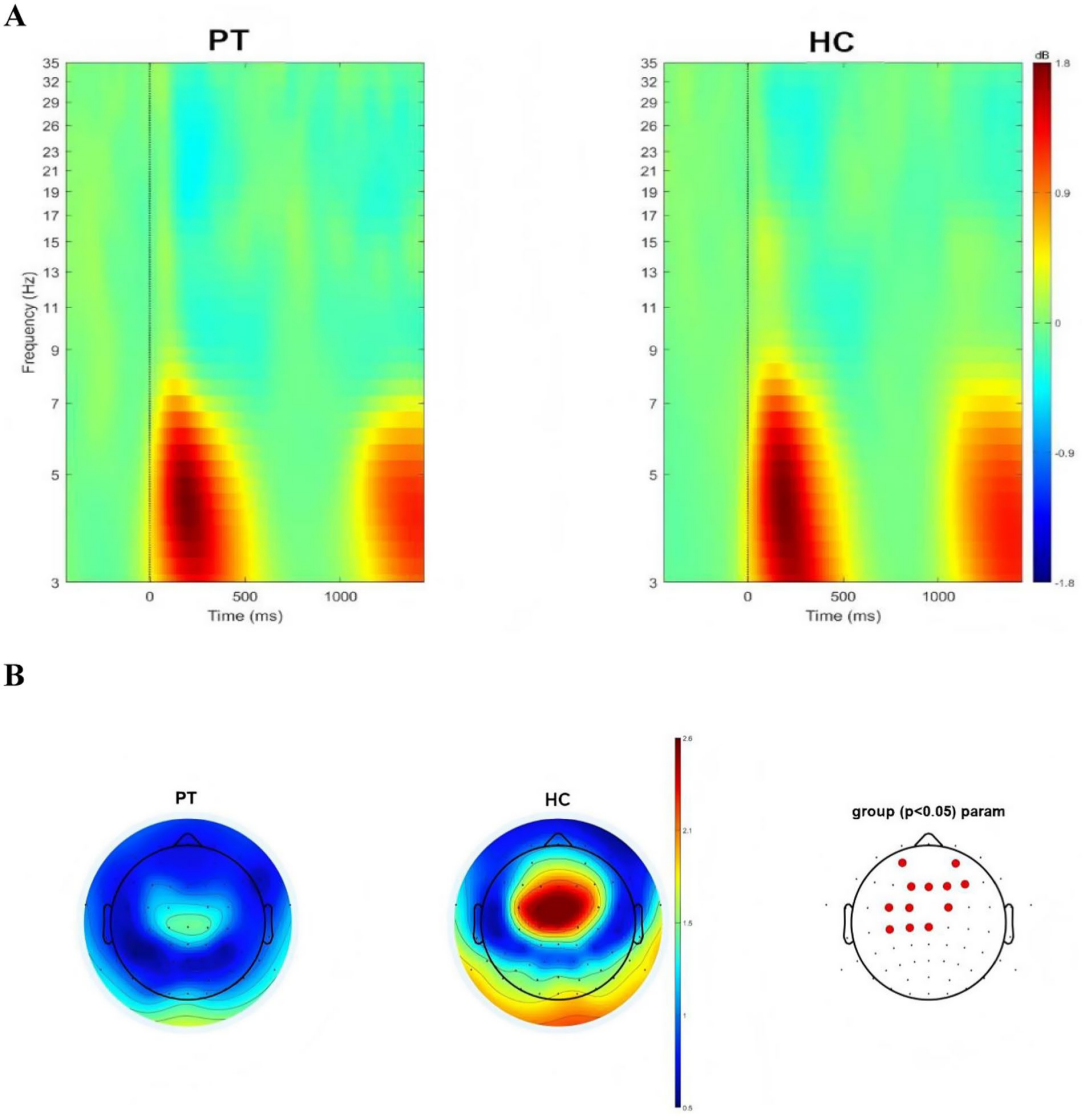
**(A)** Neural oscillations evoked by the stimuli, significant clusters at the theta band for insular glioma patients and healthy controls. **(B)** Distribution of theta oscillation topography and electrodes with differences in theta power between insular glioma patients and healthy controls.

Whole-brain time–frequency analysis was performed to identify the stimulus-evoked neural oscillations. The results showed that significant oscillatory activity between 3 ~ 7 Hz was evoked in the patients and the HC (Figure 2A). To further investigate the evoked oscillatory dynamics of the PT and HC, we extracted theta-band (3–7 Hz) power at the 64 electrodes, and compared the differences in power values at each electrode between the two groups, FDR correction was used for repeated measurement statistical analysis, with *p* value less than 0.05 was considered significant. We found that significant difference in power values between the two groups at the frontal lobe region (AF3, AF4, F1, FZ, F2 and F4 electrodes) and central region (FC3, FC1, FC2, C3, C1 and CZ electrodes; Figure 2B).

### 3.4 Whole-brain time–frequency analysis of left insular glioma group

**Figure 3:**
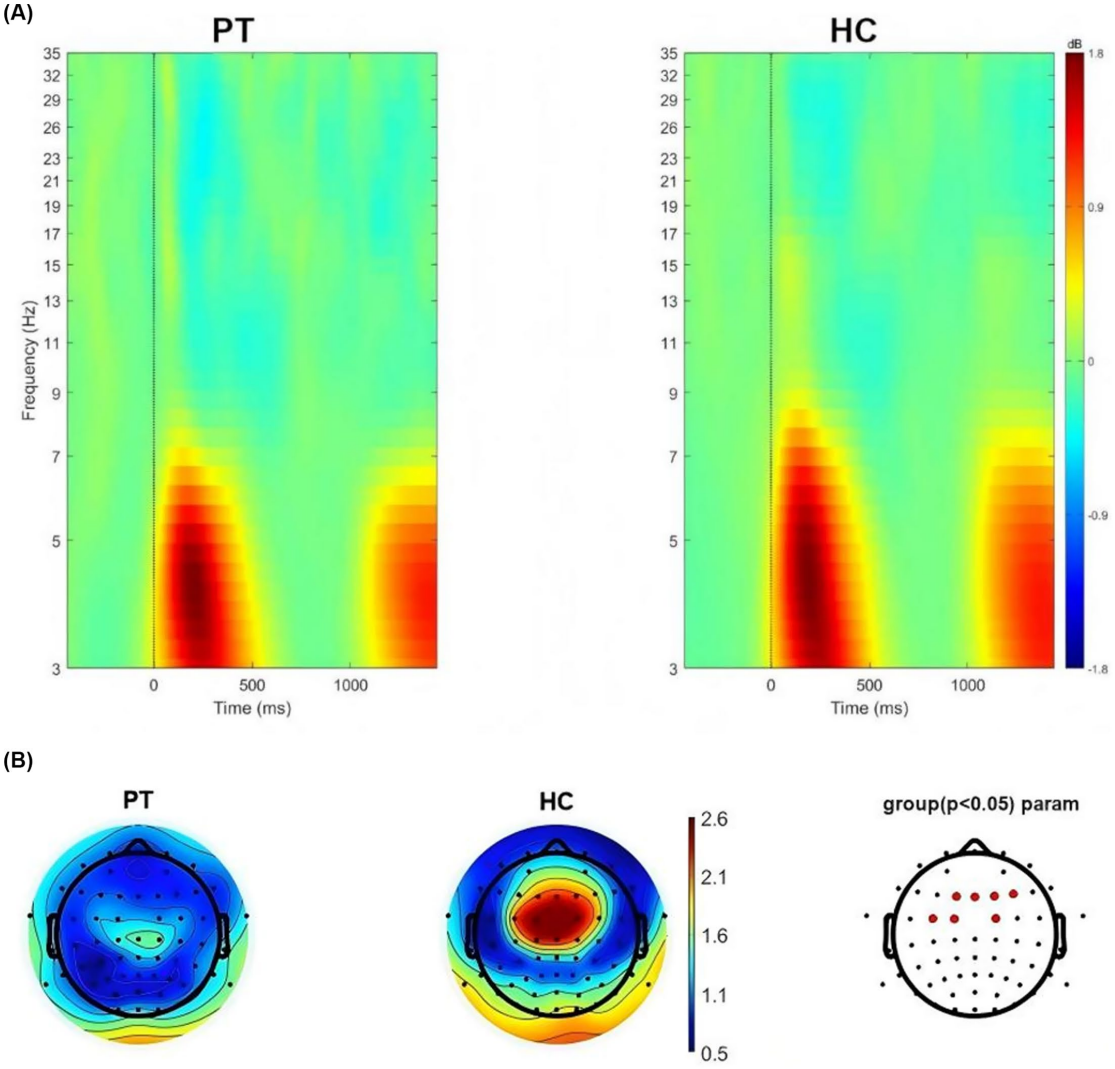
**(A)** Neural oscillations evoked by the stimuli, significant clusters at the theta band for left insular glioma patients and healthy controls. **(B)** Distribution of theta oscillation topography and electrodes with differences in theta power between left insular glioma patients and healthy controls.

We conducted time-frequency analysis on left insula glioma patients included in the study. The results showed that significant oscillatory activity between 3 ~ 7 Hz was evoked in the left insula glioma patients and the HC (Figure 3A). We also extracted theta-band (3–7 Hz) power at the 64 electrodes, and compared the differences in power values at each electrode between the two groups. FDR correction was used for repeated measurement statistical analysis, with *p* value less than 0.05 was considered significant. And we found that significant difference in power values between the two groups at the frontal lobe region (F1, FZ, F2 and F4 electrodes) and central region (FC3, FC1 and FC2 electrodes; Figure 3B).

### 3.5 Whole-brain time–frequency analysis of right insular glioma group

**Figure 4:**
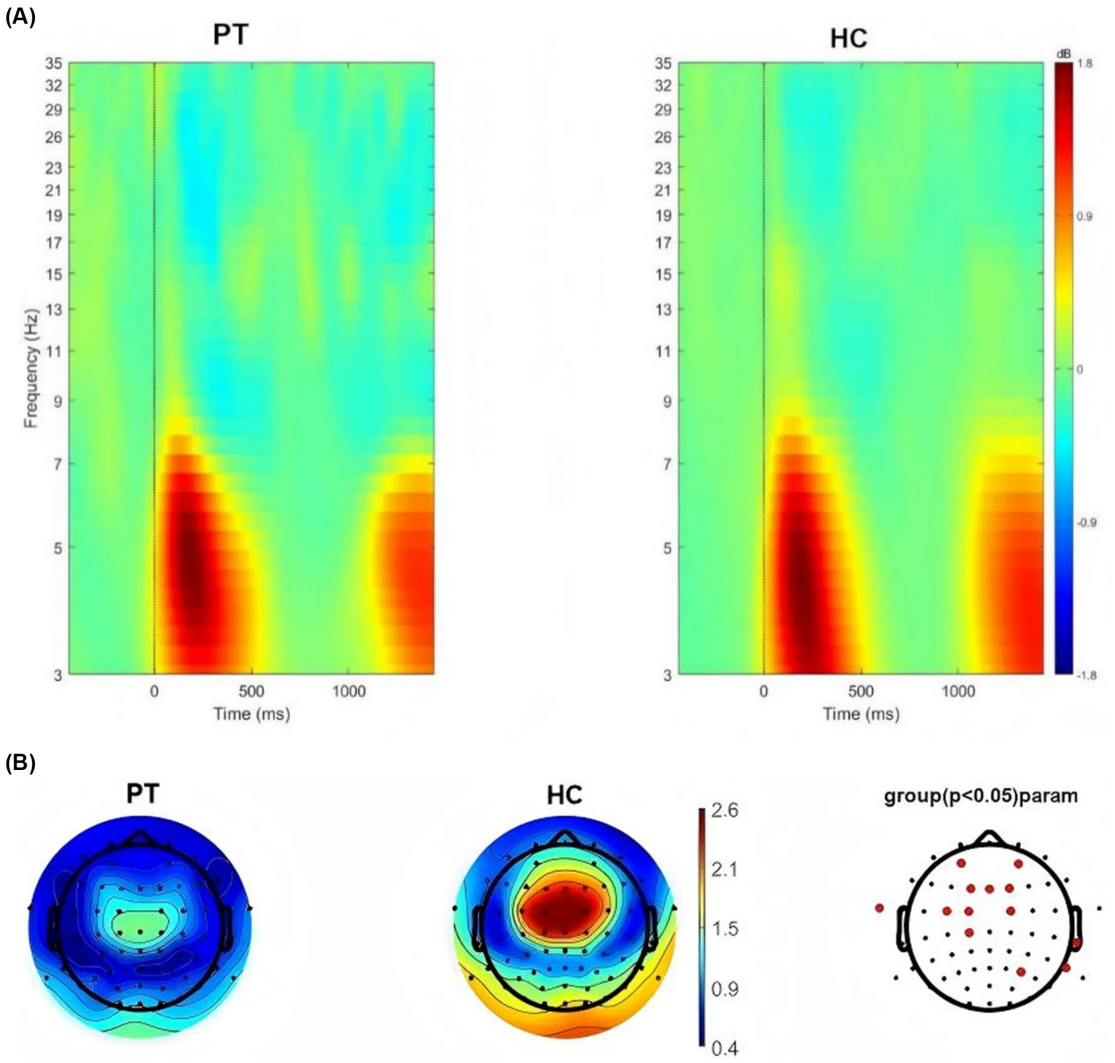
**(A)** Neural oscillations evoked by the stimuli, significant clusters at the theta band for right insular glioma patients and healthy controls. **(B)** Distribution of theta oscillation topography and electrodes with differences in theta power between right insular glioma patients and healthy controls.

We conducted time-frequency analysis on right insula glioma patients included in the study. The results showed that significant oscillatory activity between 3 ~ 7 Hz was evoked in the right insula glioma patients and the HC (Figure 4A). We also extracted theta-band (3–7 Hz) power at the 64 electrodes, and compared the differences in power values at each electrode between the two groups. FDR correction was used for repeated measurement statistical analysis, with *p* value less than 0.05 was considered significant. And we found that significant difference in power values between the two groups at the frontal lobe region (AF3, AF4, F1, FZ, and F2 electrodes), central region (FC3, FC1, FC2 and C3 electrodes) and temporal lobe region (FT9, T8, TP8 and P6; Figure 4B).

### 3.6 Correlation between the theta power and neuropsychological tests

**Figure 5:**
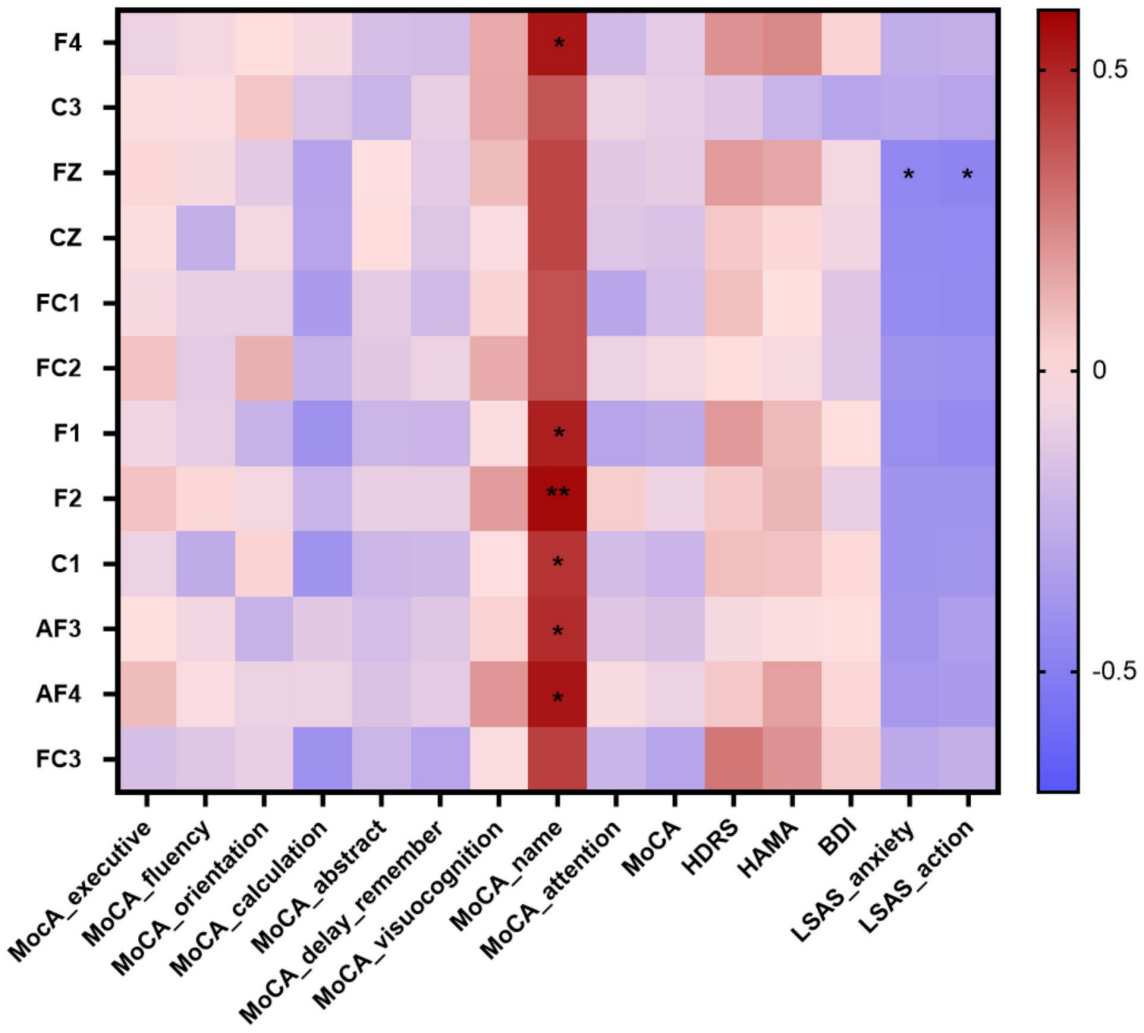
Correlation heatmap of the neuropsychological tests scores and the theta power at differentiated electrodes in patients with insular glioma. ^*^p < 0.05, **p < 0.01.

Through preoperative neuropsychological tests scores, a total of 10 patients had cognitive impairment, 18 patients had anxiety symptoms, and 20 patients had depression symptoms. We calculated Spearman’Rank correlation coefficients between theta power and neuropsychological tests scores in insular glioma patients. The theta power showed significant positive correlations with naming function at F4 (*r* = 0.539, *p* = 0.017), F1 (*r* = 0.514, *p* = 0.024), F2 (*r* = 0.572, *p* = 0.01), C1 (*r* = 0.460, *p* = 0.047), AF3 (*r* = 0.481, *p* = 0.037) and AF4 (*r* = 0.543, *p* = 0.016); showed significant negative correlations with anxiety (*r* = −0.457, *p* = 0.049) and action (*r* = −0.466, *p* = 0.044) at Fz (Table 3; Figure 5).

**Table 3:** Spearman correlation coefficient between the theta power at differentiated electrodes and the scores of the neuropsychological tests in insular glioma patients.

Indicator	F4	C3	Fz	Cz	FC1	FC2	F1	F2	C1	AF3	AF4	FC3
MocA_execution	−0.08	−0.02	0.02	−0.02	−0.04	0.08	−0.06	0.08	−0.08	0.00	0.10	−0.17
MoCA_fluency	−0.05	−0.03	−0.04	−0.25	−0.10	−0.12	−0.10	0.02	−0.27	−0.05	−0.03	−0.14
MoCA_orientation	0.00	0.07	−0.12	−0.05	−0.10	0.13	−0.23	−0.05	0.03	−0.23	−0.08	−0.09
MoCA_calculation	−0.04	−0.15	−0.31	−0.31	−0.35	−0.23	−0.40	−0.23	−0.40	−0.12	−0.07	−0.40
MoCA_abstract	−0.17	−0.23	−0.01	0.01	−0.11	−0.12	−0.22	−0.09	−0.21	−0.18	−0.16	−0.22
MoCA_delay memory	−0.18	−0.09	−0.12	−0.14	−0.20	−0.08	−0.23	−0.10	−0.20	−0.13	−0.11	−0.31
MoCA_visual perception	0.14	0.15	0.10	−0.03	0.03	0.14	−0.02	0.18	−0.01	0.04	0.20	−0.02
MoCA_name	0.539^*^	0.37	0.41	0.41	0.38	0.38	0.514^*^	0.572^*^	0.460^*^	0.481^*^	0.543^*^	0.43
MoCA_attention	−0.19	−0.08	−0.12	−0.15	−0.30	−0.08	−0.30	0.05	−0.19	−0.13	−0.03	−0.22
MoCA	−0.12	−0.10	−0.11	−0.16	−0.18	−0.05	−0.28	−0.07	−0.23	−0.17	−0.08	−0.30
HDRS	0.21	−0.13	0.18	0.06	0.09	0.01	0.19	0.06	0.09	−0.04	0.06	0.28
HAMA	0.23	−0.23	0.16	0.02	0.00	−0.04	0.10	0.11	0.08	−0.02	0.17	0.21
BDI	0.03	−0.31	−0.05	−0.06	−0.13	−0.14	0.00	−0.09	0.02	0.00	0.02	0.05
LSAS_anxiety	−0.26	−0.29	−0.457^*^	−0.44	−0.44	−0.40	−0.42	−0.39	−0.39	−0.38	−0.37	−0.28
LSAS_action	−0.25	−0.30	−0.466^*^	−0.45	−0.44	−0.41	−0.44	−0.39	−0.39	−0.34	−0.35	−0.25

*p < 0.05.

### 3.7 Correlation between P300, MMN and neuropsychological tests

**Figure 6:**
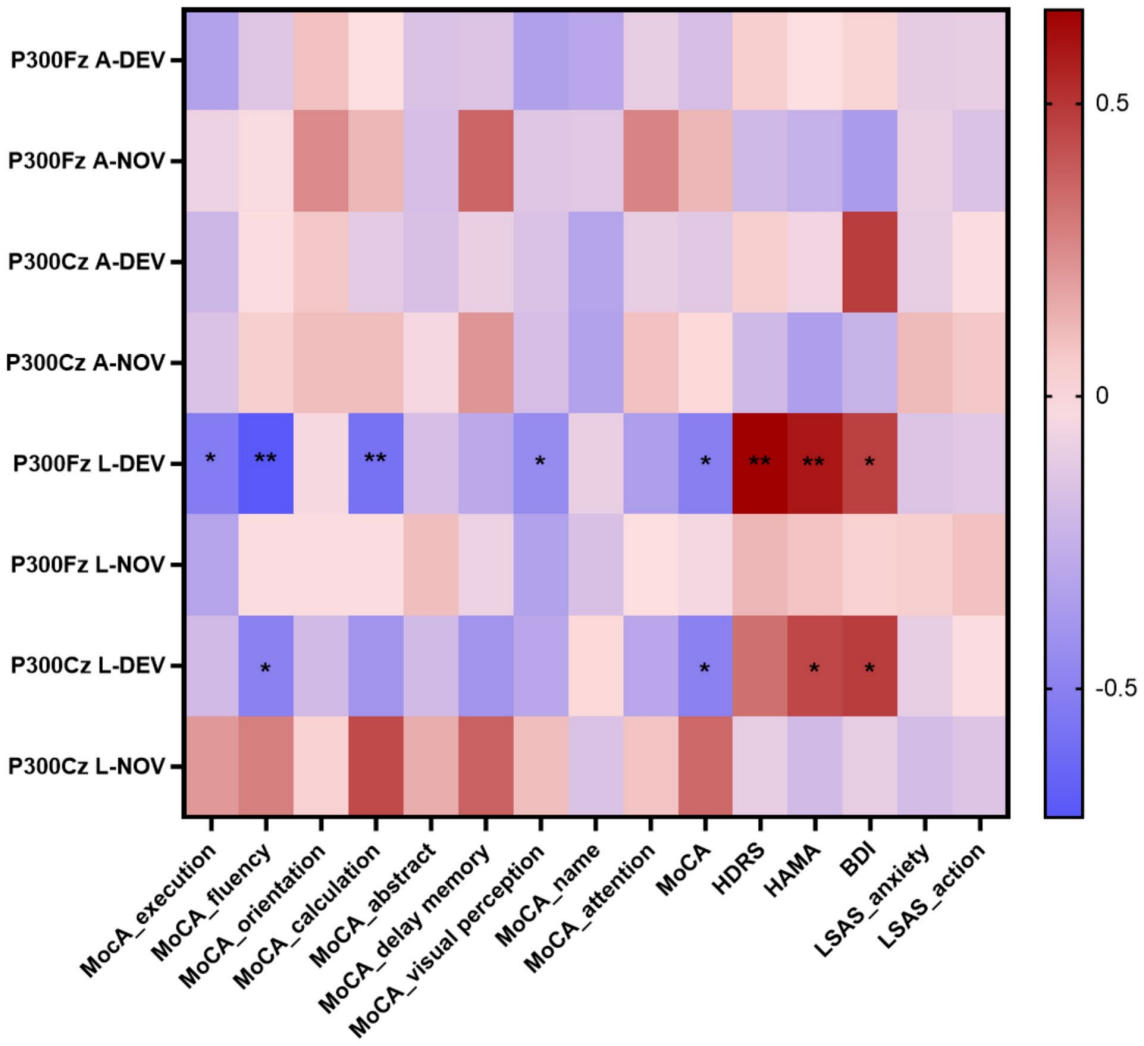
Correlation heatmap of the neuropsychological tests scores and the P300 in patients with insular glioma. *p < 0.05, **p < 0.01.

**Figure 7:**
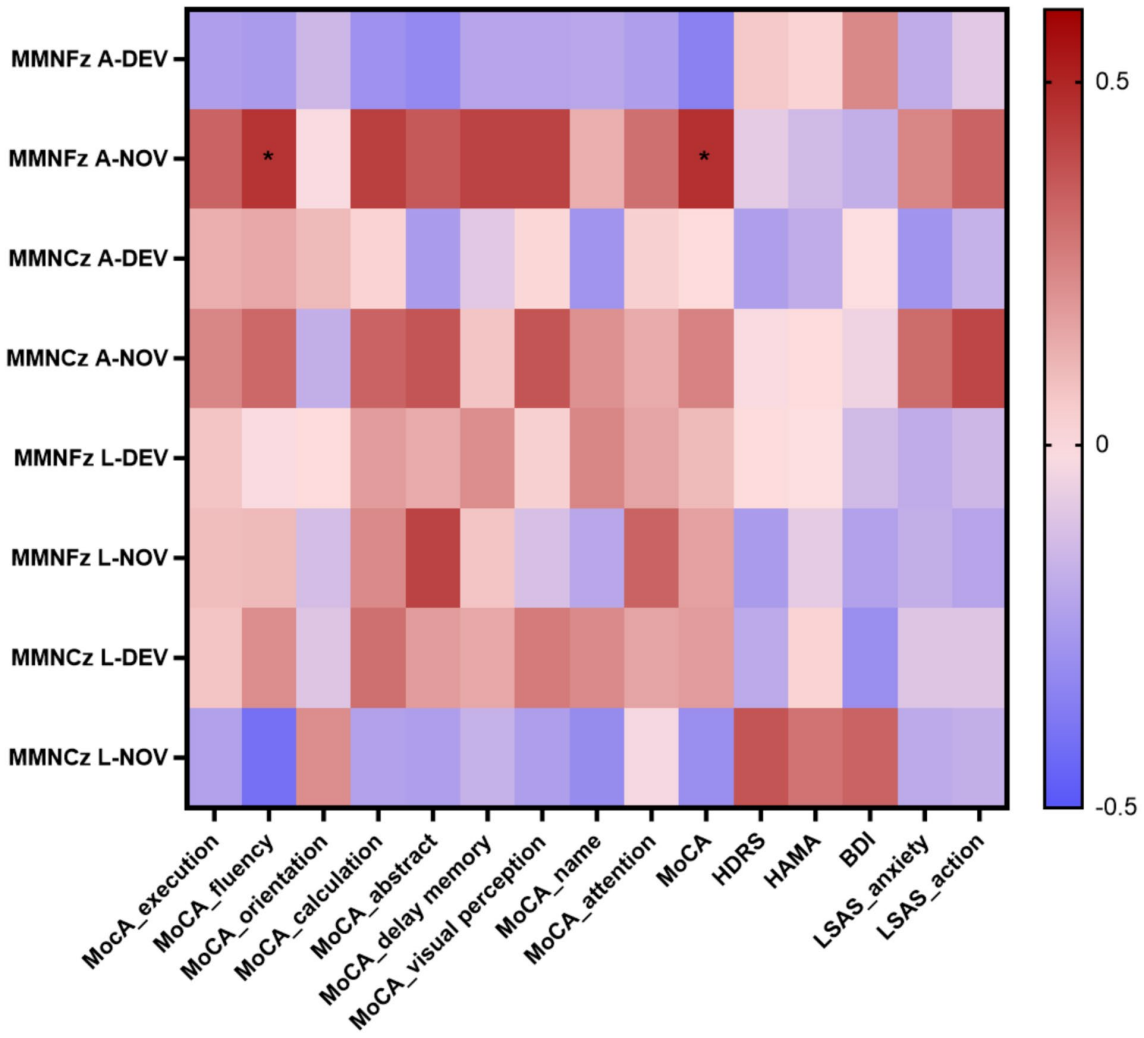
Correlation heatmap of the neuropsychological tests scores and the MMN in patients with insular glioma. *p < 0.05.

The P300 peak latency showed significant negative correlations with execution (*p* = 0.014 at Fz-DEV), fluency (*p* < 0.001 at Fz-DEV; *p* = 0.028 at Cz-DEV), calculation (*p* = 0.006 at Fz-DEV), and visual perception (*p* = 0.04 at Fz-DEV); the P300 latency showed significant positive correlations with HDRS scores (*p* = 0.001 at Fz-DEV), HAMA scores (*p* = 0.005 at Fz-DEV; *p* = 0.041 at Cz-DEV) and BDI scores (*p* = 0.036 at Fz-DEV; *p* = 0.032 at Cz-DEV; Table 4; Figure 6). The MMN mean amplitude showed significant positive correlations with fluency (*p* = 0.034 at Fz-NOV; Table 5; Figure 7).

**Table 4:** Spearman correlation coefficient between P300 and the scores of the neuropsychological tests in insular glioma patients.

Indicator	P300Fz A		P300Cz A		P300Fz L		P300Cz L	
	DEV	NOV	DEV	NOV	DEV	NOV	DEV	NOV
MocA_execution	−0.33	−0.08	−0.22	−0.16	−0.53*	−0.32	−0.2	0.21
MoCA_fluency	−0.14	−0.03	−0.02	0.05	−0.71**	−0.02	−0.5*	0.28
MoCA_orientation	0.09	0.25	0.07	0.1	−0.04	−0.02	−0.2	0.04
MoCA_calculation	−0.01	0.12	−0.12	0.1	−0.58**	−0.02	−0.4	0.44
MoCA_abstract	−0.16	−0.18	−0.17	−0.05	−0.18	0.1	−0.2	0.15
MoCA_delay memory	−0.15	0.36	−0.09	0.22	−0.29	−0.08	−0.4	0.37
MoCA_visual perception	−0.34	−0.14	−0.16	−0.18	−0.45*	−0.33	−0.3	0.1
MoCA_name	−0.3	−0.13	−0.32	−0.33	−0.09	−0.17	0.02	−0.16
MoCA_attention	−0.1	0.27	−0.1	0.09	−0.35	−0.01	−0.3	0.08
MoCA	−0.18	0.12	−0.13	0.02	−0.51*	−0.05	−0.5*	0.35
HDRS	0.05	−0.21	0.05	−0.21	0.66**	0.12	0.33	−0.1
HAMA	−0.01	−0.25	−0.06	−0.35	0.59**	0.08	0.45*	−0.2
BDI	0.03	−0.36	0.48	−0.24	0.47*	0.04	0.48*	−0.1
LSAS_anxiety	−0.11	−0.09	−0.1	0.11	−0.15	0.05	−0.1	−0.19
LSAS_action	−0.1	−0.16	−0.02	0.07	−0.13	0.09	−0.02	−0.15

*p < 0.05, **p < 0.01.

**Table 5:** Spearman correlation coefficient between MMN and the scores of the neuropsychological tests in insular glioma patients.

Indicator	MMNFz A		MMNCz A		MMNFz L		MMNCz L	
	DEV	NOV	DEV	NOV	DEV	NOV	DEV	NOV
MocA_execution	−0.24	0.33	0.13	0.24	0.07	0.09	0.07	−0.23
MoCA_fluency	−0.25	0.46*	0.15	0.32	−0.02	0.1	0.22	−0.41
MoCA_orientation	−0.15	−0.02	0.1	−0.18	0.01	−0.13	−0.1	0.22
MoCA_calculation	−0.29	0.43	0.03	0.33	0.18	0.23	0.3	−0.23
MoCA_abstract	−0.32	0.36	−0.25	0.37	0.14	0.42	0.18	−0.24
MoCA_delay memory	−0.22	0.42	−0.09	0.07	0.22	0.07	0.15	−0.17
MoCA_visual perception	−0.22	0.42	0.02	0.37	0.04	−0.12	0.27	−0.24
MoCA_name	−0.21	0.13	−0.28	0.21	0.24	−0.21	0.23	−0.31
MoCA_attention	−0.24	0.3	0.04	0.14	0.16	0.33	0.16	−0.03
MoCA	−0.35	0.47*	0.01	0.25	0.1	0.17	0.18	−0.3
HDRS	0.06	−0.08	−0.24	−0.02	0.01	−0.25	−0.2	0.37
HAMA	0.03	−0.14	−0.19	0.01	−0.01	−0.08	0.03	0.29
BDI	0.231	−0.18	−0.01	−0.05	−0.14	−0.23	−0.3	0.33
LSAS_anxiety	−0.19	0.24	−0.28	0.31	−0.19	−0.18	−0.1	−0.2
LSAS_action	−0.09	0.33	−0.17	0.41	−0.15	−0.22	−0.1	−0.18

*p < 0.05.

## 4 Discussion

In this study, we compared the differences of AERPs in insular glioma patients and health control subjects and explored the correlations between AERPs characteristics and neuropsychological tests scores in insular glioma patients. We found that P300 mean amplitude of insular glioma patients induced by novel stimuli at the Fz electrode was significantly lower than that of the HC and MMN peak latency of insular glioma patients at the deviant stimuli Fz electrode were longer than that of the HC. The P300 peak latency at Fz electrodes under deviant stimuli showed significant negative correlations with execution function, fluency, calculation, and visual perception; and it showed significant positive correlations with HDRS scores, HAMA scores and BDI scores. The P300 peak latency at Cz electrodes under deviant stimuli showed significant negative correlations with fluency; and it showed significant positive correlations with HAMA scores and BDI scores. The MMN mean amplitude at Fz electrodes under novel stimuli showed significant positive correlations with fluency. Compared to the HC subjects, the event-related theta power at frontal lobe region (AF3, AF4, F1, FZ, F2 and F4 electrodes) and central region (FC3, FC1, FC2, C3, C1 and CZ electrodes) are significantly lower in insular glioma patients. For patients with left insula glioma, the event-related theta power at the frontal lobe region (F1, FZ, F2 and F4 electrodes) and central region (FC3, FC1 and FC2 electrodes) are significantly lower than HC subjects; for patients with right insula glioma, the event-related theta power at the frontal lobe region (AF3, AF4, F1, FZ, and F2 electrodes), central region (FC3, FC1, FC2 and C3 electrodes) and temporal lobe region (FT9, T8, TP8 and P6) are significantly lower than HC subjects. Theta power at F4, F1, C1, AF3, and AF4 electrodes is positively correlated with naming function, while theta power at Fz electrode is negatively correlated with anxiety and action. Therefore, the P300 peak latency, MMN mean amplitude and event-related theta power may could be used as biological markers of the cognitive status associated with insular glioma and we may be able to monitor the progress of cognitive dysfunction of insular glioma patients or the degree of postoperative recovery in insular glioma patients by measuring these neurophysiology indicators.

The AERPs serve as a key metric for assessing the rate of spatial information processing within extensive neural networks, rendering them particularly invaluable in evaluating cognitive impairments. This is especially true in cases of neurodegenerative and neuropsychiatric disorders that are accompanied by cognitive deficiencies (23). The oddball paradigm stands out as the most frequently employed framework for analyzing cognitive processes and detecting alterations in brain activity (24). Within this paradigm, infrequent stimuli are distinguished from regular ones, triggering corresponding neural response components. Among these components, P300 and MMN are the most extensively studied AERPs. P300, the most prominent late-latency AERP component, is frequently analyzed in cognitive research and is associated with various cognitive processes, including attention allocation, working memory updating, and perceptual discrimination (10). In contrast, MMN typically signifies the automatic process of detecting deviations, reflecting the brain’s innate capability to recognize irregular inputs (11). Theta oscillations are linked to the updating of working memory, the monitoring of conflicts, and numerous other cognitive control processes. Additionally, they are involved in functional inhibition, which supports the proper functioning of executive functions (25, 26).

To our knowledge, this is the first study to compare the AERPs characteristics between insular glioma patients and health control and explore the correlation between neurophysiology indicators and neuropsychological tests results. Previous research has harnessed ERP technology to characterize ERP parameter features across a spectrum of neurological and psychiatric disorders. For instance, studies have reported decreased P300 amplitudes in depressive patients compared to healthy controls, as well as delayed P300 latencies in individuals with major depression (27). In schizophrenic patients, research has demonstrated longer latencies and smaller amplitudes of P300 compared to healthy individuals (28). Furthermore, patients with Lewy bodies and vascular dementia have exhibited prolonged latencies and reduced amplitudes of P300 (29, 30). In the context of Parkinson’s disease, Tsuchiya et al. observed decreased P300 amplitudes in frontal electrodes, whereas Ebmeier et al. reported increased MMN latencies (31, 32). In Multiple Sclerosis, Jung et al. noted decreased amplitudes and prolonged latencies for both P300 and MMN among MS patients (33). In Amyotrophic Lateral Sclerosis, Raggi et al. found decreased amplitudes and prolonged latencies in both P300 and MMN when novel stimuli were subtracted from standard stimuli in patients (34). Additionally, Beste et al. reported that patients with Huntington’s Disease who exhibited motor symptoms displayed MMN with greater amplitude and shorter latency (35). In patients with traumatic brain injury and mild head injury, Kaipio observed increased amplitudes in MMN and the late portion of P300, which was interpreted as indicating hyper-reactivity in involuntary attention mechanisms and abnormal distractibility (36). In the study of neural oscillation in prolactinomas patients, in Go trials, a negative correlation was identified between theta power and participants’ reaction times while a positive correlation was observed between theta power and the hit rate. In the Nogo condition, individuals exhibiting stronger theta activity demonstrated a reduced tendency to make false alarms (22). In the study of event-related neural oscillations in glioma patients under propofol sedation, the MCI group demonstrated a higher increase in frontal theta-ERSP during light and deep sedation than the non-MCI group did (5).

The P300 component of the ERP has been extensively linked to various conscious activities, including the allocation of attention, sustained attention, working memory updating, and the classification of information (7–10). Prior research has conclusively shown the involvement of the insula in the generation of the P300 during an oddball paradigm (37). Furthermore, the insula plays a pivotal role within the core network, which is deemed crucial for maintaining activity during continuous cognitive and behavioral tasks. It is also likely implicated in target detection tasks (38). The P300 latency reflects the processing efficiency of the prefrontal-insular network in response to stimuli (39). When the functional impairment of this network caused by insular glioma is relatively minor, cognitive resources can be allocated relatively efficiently to cognitive tasks, resulting in a shortened P300 latency. Conversely, when the network damage is severe or task difficulty increases, the P300 latency prolongs. Our study revealed that insular glioma patients exhibited longer P300 latencies compared to healthy controls, which suggests that insular gliomas exert a significant destructive impact on cognitive function and core networks, resulting in prolonged P300 latencies. Generally, there is a negative correlation between P300 latency and mental efficiency, meaning that shorter latencies are indicative of superior cognitive performance on neuropsychological tests (40, 41). Our study found that the P300 peak latency of insular glioma patients at Fz and Cz under deviant stimuli have negative correlation with the scores of certain items in MOCA test, which confirms the viewpoint of Houlihan et al. Furthermore, we found the P300 peak latency at Fz and Cz under deviant stimuli have positive correlation with the score of anxiety and depression assessment scale, which indicates that the P300 latency can not only reflect psychological efficacy, but also reflect mental health status of insular glioma patients. This may be because under anxious or depressive states, hyperactivation of the limbic system occupies fronto-insular resources, leaving insufficient resources to support efficient cognitive processing (42). This consequently leads to a prolongation of the P300 latency, with the degree of prolongation increasing in tandem with scores on emotional rating scales. However, our research did not find any correlation between the P300 latency under novel stimuli and neuropsychological tests, which may be because of the brain’s distinct processing patterns for pure tone and semantic stimuli, where familiar semantic information (novel stimuli) attracts more attention and cognitive resources so as to contribute to better psychological efficacy. In addition, we found the MMN mean amplitude at Fz under novel stimuli have positive correlation with fluency. MMN objectively reflects the automatic processing of auditory information in the brain and serves as a proxy for auditory memory integrity. The MMN amplitude can be considered as a measure of central nervous system activity, and novel stimuli attracts more attention and cognitive resources, so as to leading higher amplitude under novel stimuli. In our study, the absence of a significant negative correlation between MMN and emotional rating scales suggests that MMN is less sensitive to emotional states compared to P300. This may also be related to the automatic processing characteristics of MMN—its generation relies more on underlying sensory memory rather than fronto-insular resources (43).

Moreover, we found that compared to the HC subjects, the event-related theta power of insular glioma are significantly lower than that of the HC subjects and the theta power showed significant positive correlations with naming function, which reflected the impairment of working memory and cognitive control abilities of insular glioma patients. Due to the fact that the insula is located in an important anatomical position and is a critical node in the brain network, it is closely related to language function. The insula is involved in the rapid retrieval and naming process of vocabulary, and may result in naming aphasia after injury. In our study, the event-related theta power was positively correlated with naming function, which may be due to the fact that insula less damaged by glioma retain more complete language and cognitive functions, leading their higher event-related theta power.

Our study suggest that P300 peak latency, MMN mean amplitude and event-related theta power could be used as biological markers that indicate impaired neuropsychological functions in insular glioma patients. Based on the limitations of using only cognitive scales to assess patients’ cognitive levels, the combination of P300, MMN, event-related theta power and neuropsychological tests can be used as a more effective cognitive levels assessment tool for insular glioma patients.

There are also limitations in this study. First, the sample size is relatively small, and a larger sample size will be need in the future to validate our conclusions; Second, the cognitive scales we used are all brief screening scales, and detailed cognitive threshold scale evaluations were not performed. Third, we focused on insular gliomas and lack AERPs information of glioma patients with other locations, whether these AERPs characteristics are applicable for cognitive assessment of gliomas in other brain areas still needs to be explored. We will include glioma patients with other locations in our study to explore whether our conclusions are applicable to all glioma patients in the future.

In summary, the combined application of P300, MMN, event-related theta power and neuropsychological tests may become an optimized solution for cognitive assessment of patients with insular glioma. In the future, personalized cognitive monitoring models based on multimodal assessment (scales, electrophysiology and imaging) can be explored to improve the accuracy of disease management.

## Data Availability

The data analyzed in this study is subject to the following licenses/restrictions: patient privacy and ethical restrictions. Requests to access these datasets should be directed to xiejian0630@126.com.
